# About Digitalisation and AI, Data Protection, Data Exchange, Data Mining—Legal Constraints/Challenges Concerning Sleep Medicine

**DOI:** 10.1111/jsr.70044

**Published:** 2025-03-19

**Authors:** Bernd Feige, Fee Benz, Raphael J. Dressle, Dieter Riemann

**Affiliations:** ^1^ Department of Psychiatry and Psychotherapy Medical Center—University of Freiburg Freiburg Germany; ^2^ Faculty of Medicine University of Freiburg Freiburg Germany

**Keywords:** artificial intelligence, classification, data protection, polysomnography, sleep medicine

## Abstract

The revolution of artificial intelligence (AI) methods in the scope of the last years has inspired a deluge of use cases but has also caused uncertainty about the actual utility and boundaries of these methods. In this overview, we briefly introduce their main characteristics before focusing on use cases in sleep medicine, discriminating four main areas: Measuring sleep state, advancing diagnostics, advancing research and general advances. We then outline the current European legal framework on AI and the related topic of data sharing.

## 1 Introduction

The last few years have seen a staggering development in the utility of so‐called artificial intelligence (AI) methods specifically through the training of large language models (LLMs) on all human‐generated texts available in digital form—digitised books, newspaper and scientific articles, as well as website content and social media posts. Although the actual advances with the increasing size of the models were gradual if measured technically, rather than showing sudden ‘emergent’ abilities (Woodside 2024), at some point a threshold was crossed at which human observers would see LLM responses to human prompts or questions as reasonable and sensible. This stunning result was quickly publicised and today multiple models are widely available, both with closed and open development models. Open models generally allow unrestricted use, scrutiny and participation by anyone. Importantly, the models have also gained multimodality: Textual information can be linked to images, for example, but also to sounds and potentially any measurement modality of which sufficient data is available. These and other AI methods have clear uses in the medical field, which we will outline in the following specifically for sleep medicine.

## 2 Characteristics of AI Methods

At the basis of the recent advances in AI are neural networks (NNs), AI and NN are often used interchangeably in contemporary literature, although NNs are principally only one possible means towards the broader aim of emulating human cognitive abilities.

NNs are simplified computer models of interconnected neurons, hence the name. The simplification is twofold: The simulated neurons are strictly organised in layers between an input and an output layer, and each neuron is modelled as a simple operation on its inputs, usually summing the inputs with individual weights to form an output. ‘Deep’ NNs consist of many layers. The input weights of each neuron are typically initialized to random values in an untrained network, and during supervised training, many data examples are iteratively fed to the input layer, and the result of the output layer is compared to the associated value to be learned. The difference between the current and desired output is used to adjust the network weights by a very small amount in each presentation by ‘backpropagation’, that is, progressing from the output backwards through the layers. Training is generally performed for a fixed (large) number of iterations or until a convergence criterion is reached, in the form of a threshold for the difference between the actual and desired output for a test set of examples.

This method needs many different examples of the same output to be able to generalise: If a small set of inputs is always linked to the same output, the usually large degree of freedom of the model—one weight for all inputs of all neurons—leads to learning the concrete examples (‘overfitting’). This means that the resulting model gives the right answer to the training set but cannot sufficiently generalise to examples that are not in the training set. The need for variations in training data is often larger than the available data; in this case, ‘augmentation’ is used to create artificial variations from the training data by adding noise in those properties expected to vary in actual data.

These basic mechanisms already show that the process is likely not deterministic: Depending on starting weights and the sequence of training data presentation, many different combinations of network weights with similar performance on the test data may emerge from the training (Wortsman et al. 2021). Since these are effectively different networks, their behaviour at the ‘seams’ between the clear data examples will be different.

Another observation is the ‘black box’ characteristic of NNs: Despite good performance in recognition or prediction, the ‘rules’ the network implicitly learned are ingrained in the distributed weights and cannot be easily extracted. A whole area of AI research is dedicated to the exploration of ways to analyse trained models to extract such rules—for example, which are the most relevant clinical parameters or parameter combinations for a given diagnosis.

Thirdly, the outlined approach of presenting vast numbers of individual examples of raw data has its limitations. In the real world, many single observations of the past are no longer available in their raw form but have been aggregated into mean values and standard deviations, for example, or captured as typical cases with associated expert‐devised discriminative rules and procedures in textbooks. Another area of AI research is concerned with how to make such data available for NN training, often by generating large amounts of surrogate data from the published mean or typical data.

However, the problem of non‐determinism of each concrete trained model remains, despite possibly satisfactory performance. If many networks with similar performance are possible, which one should be chosen to form a standard?

In other words, in comparison to an explicitly formulated set of rules, trained NN models are always practically unfinishable and ephemeral, inheriting the ambiguity of the process with which they were trained. This applies to NNs in general. LLMs share this characteristic: They clearly reflect the intellectual state of the era on whose utterings they were trained and are notoriously hard to make predictable or even align to a basic set of values (Betley et al. 2025). They can still be useful if this is properly considered.

## 3 Measuring Sleep State

The current gold standard for measuring sleep state is polysomnography (PSG) with a set of staging rules designed by human experts for execution by humans (Iber et al. 2007). Particularly in the insomnia field, in which large discrepancies are observed between subjective and objective (PSG) sleep duration, the ability of AASM PSG staging to accurately reflect sleep state is disputed (Frase et al. 2023; Stephan and Siclari 2023; Feige et al. 2023).

Numerous machine learning/NN approaches have been used in the last decades, and some are already in routine use to lift the burden of staging from human raters (Stephansen et al. 2018; Yuan et al. 2019; Mousavi et al. 2019; Perslev et al. 2021; Olesen et al. 2021). An exciting development resulting from this research is hypnodensities (Stephansen et al. 2018; Bakker et al. 2023): Although in classical staging, every (usually 30 s) epoch is assigned one sleep stage, hypnodensities capture the variability across different (human) stagers and, therefore, the uncertainty inherent in the staging rules for a given PSG epoch. For NNs, it is common practice to have an output actually reflecting the probabilities of each output choice; for example, a particular epoch could be rated as 70% wake, 20% REM and 10% N1. When a ‘one‐of‐N’ choice is required, as in classical staging, the last operation in the network is a ‘softmax’ meaning that the stage with maximum probability is chosen. However, the probability output of a NN properly trained on PSG staging results by humans (ideally multiple stagers for the same data) captures the variability or uncertainty between the human stagers and, due to the generalisation properties of the network, can also determine a correlate of this uncertainty in unknown data. This uncertainty adds a finer level of distinction to PSG staging, which could result in more reliable statistics and be an interesting target for analysis with regard to sleep physiology in itself.

Despite the ephemeral nature of NNs discussed above, NNs that have been developed towards a reasonable correspondence to human raters can be used to speed up sleep staging. However, NNs learn according to the proportions of data occurring in the training set, meaning that frequently occurring combinations of sleep stages and PSG properties are learned much better than rarely occurring ones (class imbalance problem, Khan et al. 2024). In most populations of sleepers, insufficient discriminatory power for such rare occurrences leads to a small error rate overall, but sleep labs specialising in detecting sleep disorders could still be better served by human experts doing the staging.

Finally, it would be very desirable to have a ‘machine‐readable’ set of rules to replace the made‐for‐humans staging guideline. This set of rules could be designed to validly assess corner cases and eliminate the unsatisfactory inter‐rater reliability of about 0.76 (Cohen's kappa, Lee et al. 2022) observed with the current guidelines. Outside of NN‐based approaches, there have been numerous attempts to derive minimal feature sets to construct classifiers (Krakovská and Mezeiová 2011; Koch et al. 2014; Vallat and Walker 2021).

How could AI help with this? We would need the reverse process of NN learning described above, where rule‐based artificial data is created to ingrain the rules in a network: Find the minimal set of rules that reproduce all knowledge represented within a network (or the original data).

This means that the methods of AI and NNs may help with the construction of the machine‐readable set of rules, but these would probably not take the form of a NN themselves.

## 4 Advancing Diagnostics

As for PSG, NNs that are trained on large amounts of existing cases may be used to accelerate routine diagnostic tasks. In the future, a sufficient amount of data could help to further objectivise the diagnostic system of sleep and associated disorders as well as lead to optimised personalised treatments.

There is, however, a severe lack of clinical data for this task. Although there are sleep labs that routinely store their raw PSG data and therefore accumulate information that may be used to train the sleep staging systems and also the sleep diagnostic systems of the future, the EU General Data Protection Regulation (GDPR; see below) created a significant obstacle to using this personal and medical condition‐related data on a general basis and between labs. Secure and trusted ways to put this data to use are urgently needed (see below).

Clinical data in general are even harder to obtain because of the pass‐through character of most clinics and practices. Systematic digital data is typically only recorded where mandated or necessary for billing and are not organised and stored in a common fashion (Edmondson and Reimer 2020; Declerck et al. 2024). National patient registers, such as the one in Denmark, have proven to deliver invaluable results, especially regarding long‐term real‐world effects of treatment (e.g., Jennum et al. 2024, 2025 for ADHD treatment and CPAP), disease correlates (e.g., Framke et al. 2024 for sleep disturbances in Multiple Sclerosis) and predictors (e.g., Gronemann et al. 2020 for risk factors of treatment‐resistant depression). These are most valuable for evaluating the disease burden of the population, assessing the general efficacy of treatments and consequently steering the healthcare system as a whole.

Another well‐known example of the success of data availability is the UK Biobank (Allen et al. 2014). Although this was not a mandatory state‐wide collection as in Denmark, subjects registered with the National Health Services (NHS) were asked for their participation and informed consent. This allowed a huge set of questionnaire and imaging data, as well as biosamples and derived biomarkers, to be assessed and has proven to be another source of great value, particularly regarding genomic, plasma protein and multimodal imaging markers. Although the information on sleep quality in the UK Biobank is limited, the data did enable valuable insights (e.g., Kyle et al. 2017; Ell et al. 2023).

As noted above, one problem with using clinical data is the lack of a common standard for reporting and storing it. LLMs are designed to ‘make sense’ of data and have recently been used to bring clinical data from heterogeneous sources into a common format (Kirchler et al. 2025). Since it will probably be impossible to enforce reporting rules worldwide and single endeavours, even of unprecedented scope, will always be limited and not include all the data collected over many years prior to standardisation, using AI in this ‘data preparation’ phase will be important to build a sufficient database. Additionally, as noted above, prior evidence condensed into aggregated values (as published in studies) and into expert rules and opinions has to be used as well and methods are developed for this. As AI systems are continuously evolving, this will be an iterative approach with new methods applied to the available raw data in each step.

## 5 Advancing Research

Research, like diagnostics, profits from the acceleration of standard tasks and increased data availability (cf. ‘General advances’ below). More specific uses, always considering the ephemeral nature of each trained model, are often linked to the complex categorisation and detection abilities of AI methods. In many cases, this opens up whole research areas.

For example, voice analysis can be used for emotional prosody and mood detection (Stassen et al. 1995; Larrouy‐Maestri et al. 2024). Similarly, facial emotions can be detected and included in the research agenda (Baltrušaitis et al. 2018; Liu et al. 2025).

Specifically in the sleep field, hypnodensities (Tracey et al. 2024) have already been noted above. Hypnodensities provide novel insights into staging uncertainties. It is possible that epochs during which the sleep stage is uncertain actually contain intermediate or mixed brain states, possibly in the sense of ‘local sleep’ (ElGrawani et al. 2024). At the very least, hypnodensities show where the most common fallacies of our current staging rules lie and where our concepts about sleep and the staging rules could be amended.

Also, the study of sleep can be extended in multiple directions, deriving measures of sleepiness from speech or video recordings of subjects, deriving sleep state from other electrode configurations like circumaural configurations (Mikkelsen et al. 2019), or different channels (Van Der Aar et al. 2023) or other modalities altogether, like ECG and actigraphy (Thiesse et al. 2022) or ‘smart garments’ (Tang et al. 2025). In animal research, an example is sleep staging through wide‐field calcium imaging data in mice (Zhang et al. 2024).

With Research Domain Criteria (RDoC; Insel et al. 2010), a framework or coordinate system has been established 15 years ago to base mental health research on quantifiable axes instead of man‐made diagnostic categories. A central problem with this approach is that diagnostics define disease and need for treatment as well as treatment success, meaning that studies on diseases and treatment will necessarily work within diagnostic boundaries. Newer accounts acknowledge this and note that RDoC will ‘inform’ future diagnostic systems (Morris et al. 2022; McGorry et al. 2025) and also propose a further evolution of RDoC itself (Quah et al. 2025). Since pure transdiagnostic research is hard to do, AI methods can help to build the necessary data by retrospectively classifying evidence into suitable coordinates. This has been done on grant abstracts (Perlis 2025) but it can also be used to categorise results from published studies and conduct meta‐analyses. Furthermore, LLMs can be used to transform existing clinical notes to estimates of RDoC dimensions (McCoy and Perlis 2024).

## 6 General Advances

In the advances discussed above, it has already become clear that making good use of AI methods often involves facilitating diagnostics and research in a general and indirect way.

Communication with patients, for example for consent management (Mirza et al. 2024) and questionnaires, can be supported by chatbots (Olawade et al. 2024), using textual or even voice interaction rather than screen or paper forms. The chatbots can ask back if responses are unclear or provide additional information until informed consent is truly reached and questionnaires are properly filled. This could be used, for example, to make high‐density sampling of subjective sleep continuity for insomnia in the form of sleep diaries (Perlis et al. 2025) less tedious and more reliable.

Data can be acquired from sources too tedious to work on by human data entry workers, by scanning notes and medical reports and using AI methods to automatically recognise important data and organise it in tabular form.

Researchers can be supported with programming and data analysis tasks—from support while developing their own software, to writing software given a task description, to interactive data analysis and visualisation without needing to program at all (e.g., Wang et al. 2024).

All of these uses contribute to higher throughput, increased productivity and increased data availability, allowing advances in diagnostics and research.

## 7 AI and Data Sharing Regulations in the European Union

It is obvious that large quantities of data are necessary to allow NNs to derive ‘knowledge’ from the data. At the same time, sleep data—particularly the PSG raw data that is especially interesting for automated analysis methods—provides rich person‐related information that is linked to medical conditions and is, therefore especially, protected by the GDPR (2016) rules of the European Union. Article 9 prohibits the processing of such data, subsequently allowing its use only in specific exceptions for legitimate uses. Obvious exceptions include the use necessary for the diagnosis and treatment of medical conditions within the scope of the treatment contract. The only broadly applicable exception is data use to which the data subject gave explicit informed consent. Other exceptions are possible if the data are necessary to reach an aim of substantial public interest and the effort to obtain individual consent would be prohibitive for that aim. Due to substantial uncertainties regarding this regulation, the EU has devised additional ‘procedural rules’ to clarify and harmonise the application of the GDPR (GDPR procedural rules 2025). Even if these rules will probably allow the use of ‘historical’ medical data without retrospectively obtaining individual consent, it is important that the basic rights of the data subjects must still be respected, represented by ethics and data protection boards: This is particularly achieved by heeding data protection standards during transmission and storage, by destroying data copies after the agreed project time, by limiting data use to the agreed purpose and by limiting data access to defined entities bound by these requirements.

Also, for current and new research projects, obtaining the data subject's informed consent is necessary, which is the reason for large efforts to develop standardised consent instruments. In Germany, the ‘Broad Consent’ was developed but criticised for being too general, making it difficult for data subjects to know who has their data and for what purpose; this is why web‐based consent management and information hubs are being developed (Forschen fuer Gesundheit 2023; Burmeister et al. 2024).

Special considerations apply when medical data are used for AI training. At first, training seems to require access to all raw data sets in one place. Keeping copies of data sets from multiple sources in one place poses several risks, as large data collections are interesting targets for hacking attacks, and consent, data use restrictions, deletion requests as well as data updates must be managed across sources. For these reasons and because institutions often do not allow the transfer of raw patient data to outside entities, federated learning approaches have been developed (Gruendner et al. 2019). In federated learning, the training data remain within their institutions of origin, but the model to be trained is sent to different institutions or training nodes (Rieke et al. 2020).

However, federated learning alone is not sufficient to ensure data privacy. The current gold standard is to additionally employ methods of Differential Privacy (Kaissis et al. 2021; Ha et al. 2022). The definition of privacy here is to make it sufficiently hard for an attacker to judge whether the data of a particular subject was included in a training set. This aim is reached by adding carefully calibrated noise to the data, striking a balance between privacy and model performance (Bu et al. 2020; Ziller et al. 2021).

Another avenue that is currently explored is to train NN models on encrypted data (Hesamifard et al. 2017; Khowaja et al. 2022). A promising cryptographic solution to many of the privacy problems in medical research addressed above is ‘secure multi‐party computation’ (SMPC, cf. Lindell 2020 for an introduction). SMPC allows multiple parties to learn from each other's data without actually seeing the raw data.

The EU AI Act (EU AI Act 2024) establishes a risk‐based approach to AI applications. It specifically defines prohibited AI practices as well as a high‐risk category and specifies risk management systems mandatory for these use cases. The prohibited practices (Chapter II) mostly refer to methods of mass surveillance and methods suited for oppression. High‐risk AI systems (Chapter III) mainly cover systems that autonomously decide matters of possible risk or harm to natural persons or perform profiling on them. Therefore, many medical uses would be considered high risk unless a human expert remains in the loop leading to decisions and actions. In sleep diagnostics and research, this would conceivably not be relevant.

Chapter IV, however, clearly has implications also for the sleep field: It defines transparency obligations for providers and deployers of AI systems. These obligations include making transparent that and for which purpose an AI system is being used. The aim of ‘Trustworthy AI’ calls for carefully documenting scope, capabilities and mode of use of employed AI systems. Regarding capabilities, the use of Bayesian Neural Networks (BNN; Magris and Iosifidis 2023) is a solid approach: Classical NNs do not allow any statistical assertion on their output, for example by which amount the output would change if the inputs change by a small amount (measurement error) or how sensitive the output is to small changes in some weights (training error). BNN actually learn probability distributions and therefore can document the stability of a result, which is of course essential for trusting them and base decisions on them. Although the AI Act itself only demands ‘sufficient transparency and explainability’, another important development is Explainable AI (Pahde et al. 2025). This term denotes NN models that incorporate the ability to show which features of the input data are most important for a given decision. Such systems are increasingly used in sleep medicine (e.g., Barnes et al. 2022; Dutt et al. 2023; Vaquerizo‐Villar et al. 2023; Adey et al. 2024).

## 8 Summary and Outlook

These are exciting times. The capabilities of computer systems are ever increasing and facilitate diagnostics and research, but also the surveillance and manipulation of humans in society. This is why the AI Act and GDPR are necessary. It is important to note that there are ways to employ the power of computer systems to solve the ensuing problems in a way that preserves privacy and data sovereignty in the very sensible medical field. For example, data trustee systems should be widely employed and automated. The vast number of requests that will need to be processed cannot be handled if a data access committee deliberates on every single request. Rather, the committee should establish rules which are then applied automatically, with secure digital identities and signatures that make data use contracts enforceable.

‘Data lakes’ pooling across institutions for longer periods (as opposed to the scope of a single use) may prove infeasible for medical data because of the costs of securing and maintaining them as well as documenting allowed usage. Even for electronic health records, central storage can be kept to a minimum, making directly available only health data needed in case of an emergency (blood group, relevant medical conditions). All other data, such as the most recent ECG or MRI, can be requested online, given sufficient authorization. Such data request systems for both clinical and research uses must be urgently introduced.

## Author Contributions

**Bernd Feige:** conceptualization, investigation, writing – original draft. **Fee Benz:** writing – review and editing. **Raphael J. Dressle:** writing – review and editing, investigation. **Dieter Riemann:** writing – review and editing, funding acquisition, supervision.

## Conflicts of Interest

The authors declare no conflicts of interest.

## Data Availability

Data sharing is not applicable to this article as no new data were created or analyzed in this study.
